# The Antiparkinsonian and Antidyskinetic Mechanisms of *Mucuna pruriens* in the MPTP-Treated Nonhuman Primate

**DOI:** 10.1155/2012/840247

**Published:** 2012-09-10

**Authors:** Christopher A. Lieu, Kala Venkiteswaran, Timothy P. Gilmour, Anand N. Rao, Andrew C. Petticoffer, Erin V. Gilbert, Milind Deogaonkar, Bala V. Manyam, Thyagarajan Subramanian

**Affiliations:** ^1^Buck Institute for Research on Aging, Novato, CA 94945, USA; ^2^Department of Neurology, The Pennsylvania State University College of Medicine, Hershey, PA 17033, USA; ^3^Department of Neural and Behavioral Sciences, The Pennsylvania State University College of Medicine, Hershey, PA 17033, USA; ^4^Center for Neurological Restoration, The Cleveland Clinic Foundation, Cleveland, OH 44195, USA

## Abstract

Chronic treatment with levodopa (LD) in Parkinson's disease (PD) can cause drug induced dyskinesias. *Mucuna pruriens* endocarp powder (MPEP) contains several compounds including natural LD and has been reported to not cause drug-induced dyskinesias. We evaluated the effects of *Mucuna pruriens* to determine if its underlying mechanistic actions are exclusively due to LD. We first compared MPEP with and without carbidopa (CD), and LD+CD in hemiparkinsonian (HP) monkeys. Each treatment ameliorated parkinsonism. We then compared the neuronal firing properties of the substantia nigra reticulata (SNR) and subthalamic nucleus (STN) in HP monkeys with MPEP+CD and LD+CD to evaluate basal ganglia circuitry alterations. Both treatments decreased SNR firing rate compared to HP state. However, LD+CD treatments significantly increased SNR bursting firing patterns that were not seen with MPEP+CD treatments. No significant changes were seen in STN firing properties. We then evaluated the effects of a water extract of MPEP. Oral MPWE ameliorated parkinsonism without causing drug-induced dyskinesias. The distinctive neurophysiological findings in the basal ganglia and the ability to ameliorate parkinsonism without causing dyskinesias strongly suggest that *Mucuna pruriens* acts through a novel mechanism that is different from that of LD.

## 1. Introduction

Dopamine replacement therapy with LD+DDCI (dopa decarboxylase inhibitor) is the most effective pharmacological treatment for PD. However, LD+DDCI remains expensive and out of reach of many PD patients in developing countries [1] and causes disabling drug-induced dyskinesias, motor fluctuations, and neuropsychiatric complications in most PD patients [2–4]. Development of an oral treatment with the same or higher efficacy of LD+DDCI that does not cause drug-induced complications is an unmet need.

MPEP in Ayurvedic medicine provides alleviation of parkinsonism but has been reported to not cause drug-induced dyskinesias [5–7]. MPEP contains 4-5% natural LD, which had been implicated as its main mechanism of action and the reason for not causing drug-induced dyskinesias (i.e., did not contain enough LD). Despite MPEP being well-tolerated in clinical trials [8–10], PD patients complain of inability to consume a large volume (30 g) of this leguminous protein as it often causes adverse gastrointestinal side effects. Therefore, despite wide availability of MPEP as a nutraceutical via Internet marketing and in Ayurvedic pharmacies, MPEP is rarely utilized as allopathic treatment for PD even in India [1, 11]. This lack of popularity of MPEP is related to its gastrointestinal side effects and the erroneous notion that MPEP simply represents a natural form of low-dose LD. However, MPEP is ubiquitously used by Ayurvedic physicians worldwide as an ingredient in medications for the treatment of PD.

We recently reported that a newly formulated, simple MPEP water extract (MPWE) given parenterally significantly ameliorates parkinsonism with reduced drug-induced dyskinesias in the HP rat [12]. This suggested that its anti-PD and antidyskinetic effects could not be explained by the presence of small quantities of natural LD alone and that MPWE is more effective when used alone without the addition of a DDCI. These surprising findings led us to further investigate the unique anti-PD and anti-dyskinetic properties of MPEP and MPWE in the 1-methyl-4-phenyl-1,2,3,6-tetrahydropyridine-(MPTP-) treated parkinsonian monkey. Further evaluation of the mechanistic actions of MPEP and MPWE may increase its potential as an alternative therapy for PD.

## 2. Materials and Methods

### 2.1. Animals

Fourteen adult (6–9 kg) rhesus (*Macaca mulatta*) and two cynomolgus (*Macaca fascicularis*) monkeys received either intracarotid (ICA) MPTP to induce an HP state, ICA+systemic (IV) MPTP to induce an overlesioned HP (OHP) model, or systemic (IM or IV) injections of MPTP to induce bilateral parkinsonism [13–16]. Each animal was operant-conditioned behaviorally trained [17] to accept medications to ensure proper consumption and clinical oral simulation. Clinical assessments were taken after MPTP to ensure stability of parkinsonism and at subsequent treatment exposure using the modified version of the Unified Parkinson's Disease Rating scale for primates (mUPDRS) [15]. Electrophysiological recordings before and after treatments were done in awake, behaving parkinsonian animals. Standard extracellular single-cell recording techniques were used as described in detail elsewhere [16]. All procedures were in compliance with the “Principles of Laboratory Animal Care” (NIH no. 86–23, revised 1985) and approved by the institutional animal care and use committee.

#### 2.1.1. Administration of MPTP to Induce Parkinsonism

For ICA administration of MPTP to create an HP state, animals were placed under deep general anesthesia, the left common carotid artery was exposed, and the internal carotid artery was isolated followed by manual retrograde injection of MPTP solution (0.5 mg/kg body weight at a concentration of 1 mg/mL) over a period of 15 minutes. The animal was allowed to recover and assessed for stability of HP. Depending on stability of HP state (see below for behavioral testing details), exposure to ICA MPTP was performed up to 4 times in each animal. Repeat surgeries were not performed before 2 weeks of observation had been completed and surgical scar from the previous surgery had healed. The cumulative ICA MPTP dose ranged from 0.5 to 2.5 mg/kg. A subset of animals was rendered overlesioned HP (OHP). To achieve an OHP state, animals were initially treated with ICA MPTP. Once HP state was stable, the animal received subsequent injections of IV MPTP (0.2 mg/kg), inducing mild parkinsonism in the previously unaffected side. Another set of animals was rendered bilaterally parkinsonian with systemic IV or IM injections of MPTP (0.2 mg/kg). Cumulative doses of systemic MPTP ranged from 0.2 mg/kg to 1.0 mg/kg. Drug treatments were then given only when animals were stable parkinsonian for >3 months as determined by no changes in the mUPDRS ratings (see below) performed twice each month separated by a minimum of 15 days and operant-conditioned for a minimum of 6 months such that they were compliant with oral dosing of medications (see the section below).

#### 2.1.2. Operant Conditioning for Oral Medication Compliance without Compromising Enrichment Protocols in Parkinsonian Primates

The following protocol was used for operant conditioning and behavioral training in each animal to ensure compliance and complete consumption of antiparkinsonian medications. Each animal was individually housed such that visual and olfactory contact with conspecifics was maintained at all times. Various types of toys were placed in each cage and rotated every other week. At any one time, every cage contained a hanging toy, such as a ball or a mirror and at least one (usually two) chewing toy, such as a Hercules dental chew toy, Dental ball, Kong toys, nylabones, or pieces of wood (Bio-Serv). In some instances, certain animals showed adverse stress reactions to certain types of toys. In this case, that toy was removed from the animal's cage and replaced with another toy. In addition to the regular feed, monkey diet was supplemented with fruits, vegetables, nuts, or other types of “treats” every day. The size, quantity, and time of day that these “treats” were given (always in the late afternoon after training) were monitored carefully so as not to interfere with our behavioral training. Also, each day, Monday–Friday, the animals were presented with an enrichment activity. These activities included watching cartoons, “novel” food day, foraging devices, and special activities such as air-popped popcorn or bowls of water to play with. On weekends, the animals were given extra food treats. Cages were checked for any remaining monkey biscuits or treats each evening; any remaining food was removed in order to maintain the appropriate level of food scheduling necessary for operant training of a food-picking task or voluntary consumption of medications. All animals were observed for stereotypical (pacing, rocking, digit-sucking) and self-injurious behaviors (self-biting, head banging). If any such behaviors were seen, enrichment for those animals was increased. Oral LD and MPEP were successfully administered twice daily (AM and PM) by hiding powdered drug in food treats. MPWE was typically given orally without the need of hiding it in any additional foods or liquids. Animals appeared to enjoy MPWE and voluntarily consumed it completely at each dose. Improvement in parkinsonism was then assessed by using the mUPDRS and confirmed through analysis of blood plasma levels in representative animals at the same time as the mUPDRS exams. Animals were continuously monitored to ensure complete consumption and drug compliance during treatments by investigators. Depending on the desired task (consumption of treatment or interaction with investigator to evaluate mUPDRS), any of the enrichment protocols in Table 1 could be detrimental to operant conditioning. Similarly, any of the training conditions could cause behavioral problems in monkeys due to lack of appropriate enrichment. To overcome these barriers to operant conditioning, we limited the time when enrichment was given (all enrichment was given after training session to maintain food scheduling), scheduled enrichment only in the afternoon or following the end of a testing session, and maintained visual, sound, and olfactory stimuli without interfering with operant conditioning. The same individuals were involved in the enrichment, operant conditioning, and husbandry to limit distress and to reinforce the operant conditioning. Veterinary care interaction with these monkeys with individuals other than the persons involved in operant conditioning and enrichment was limited to yearly physicals and twice a year TB testing and any other USDA mandated veterinary checkups.

#### 2.1.3. Clinical Assessment

Behavioral ratings were performed using the mUPDRS in a blinded fashion. A more detailed description can be found in our previous publications [15, 16]. Briefly, the mUPDRS consists of subjective-rater-dependent but validated and reliable blinded evaluations of vocalization/hooting, facial expression, tremor (rest or action), muscle tone/rigidity, hypokinesia, finger dexterity, foot agility, balance/postural instability, spontaneous gait, dystonia, and circling/dyskinesia. Each item on the mUPDRS has a range from 0 (no motor deficits) to 4 (very severe impairment) for each limb or body part and is modeled after the UPDRS was used to rate PD patients in clinical trials. Animals were also further assessed for drug-induced dyskinesias using a modified Abnormal Involuntary Movements Scale (AIMS) previously described for primates [18, 19]. AIMS scores are represented as the total sum of dystonic posturing and choreiform movements in the face, trunk, and each limb. Severity was evaluated using the following scale: 0 = none; occasional, mild = 1; intermittent, moderate = 2; continuous, severe = 3. The entire clinical rating session was videotaped for minimum of 4 hours after each dose of medication for a minimum of 8 hours of video in representative animals. mUPDRS and AIMS scores were taken at stable parkinsonian baseline state, placebo, and at an average time of 75 minutes after drug treatments. We established the optimal LD/CD dose for each animal using blinded testing every 2 weeks at monthly intervals starting at a dose of 50 mg LD/12.5 mg CD b.i.d. (i.e., LD 100 mg/CD 25 mg/day) and escalated by 100 mg LD every 2 weeks to achieve no further improvements in mUPDRS scores despite dose escalation. Thereafter, the lowest dose of LD that produced the largest improvement in mUPDRS scores was chosen as the optimal dose of LD in each animal. Thus, animals were tested on LD treatment for a minimum of 3-4 months to determine their optimal LD dose. All animals were washed out of LD treatments for 1 month before initiating MPEP or MPWE treatments. In a similar fashion, optimal dose of MPEP, and MPWE was also determined and optimal behavioral plateau that coincided for LD, LD+DDCI, MPEP, and MPWE was identified. This plateau phase was used for all the experiments described in this paper to make comparisons with equipoise and validity. 

#### 2.1.4. Electrophysiology

After recordings were taken during placebo and on drug treatments, the recordings were sorted by offline principal component analysis, and interspike intervals (ISIs) were generated. Acceptable records were comprised of at least 400 spikes and had duration between 60 and 120 sec. Firing rates and seven measures of the firing patterns were employed: the coefficient of variation (CV) of the ISIs, the burst index (mean of the ISI distribution divided by the mode [20]), the percent of spikes in bursts and percentage of time in bursts calculated by the Poisson surprise method [21], the density discharge histogram (DDH) compared to the DDH of a random Poisson spike train [22], the range of the DDH, and the sample entropy [23]. The seven numeric firing pattern metrics were compared using the Wilcoxon-Mann-Whitney rank-sum test, and the categorical DDH classification was compared using Fisher's 2 × 2 exact test (grouping together “Poisson” and “bursty” categories).

### 2.2. Drug Treatments

#### 2.2.1. Preliminary Dose-Finding and Toxicity Studies of MPEP: Experimental Design and Results

We first completed a preliminary dose-finding study to determine optimal doses and toxicity adverse effects of MPEP and MPEP+CD. HP monkeys were tested on MPEP and MPEP+CD (25 mg) to find optimal dosing after attaining stable HP state (*N* = 3). Each treatment epoch was followed by 2 weeks of washout. Two separate mUPDRS scores 14 days apart were obtained on placebo and for each treatment epoch. MPEP was titrated in these studies from 6 g/day to the highest dose of 18 g/day (*N* = 11) to evaluate gastrointestinal effects, drug-induced dyskinesias, or behavioral correlates of psychiatric symptoms. Blood draw was performed 90 minutes after administration and consumption of medications (placebo, LD+CD (250 mg/62.5 mg) and MPEP+CD (4.5 g/25 mg)) to test the bioavailability of orally administered LD and MPEP at approximately equivalent doses for pharmacological estimation of dopamine levels. See Table 2 for representative drug-dosing block design.

In these initial studies, mean mUPDRS scores improved by 4% (change from 35 to 33.5), 24.2% (35 to 26.5), and 27.1% (35 to 25.5), respectively, with 3 g, 6 g, and 9 g (total daily dose) of MPEP alone. Optimal dose for MPEP+CD was determined to be 9 g of MPEP + 50 mg of CD/day. In this experiment, mean mUPDRS score showed no improvements for the placebo treatments (mean mUPDRS score of 35 to 35.2) compared to a 49.1% improvement (mean mUPDRS score changed from 35 to 17.8) at this optimal MPEP+CD dose. No observable adverse events were evident at these doses of MPEP alone or MPEP+CD. Doses at 12 g/day and 18 g/day caused compliance issues and severe adverse effects with successful consumption. These included nausea, retching, vomiting, behavior that mimicked hallucinations, and increased aggression. Serum peak dose estimation of dopamine levels was 180 pg/mL after placebo treatment, 27,600 pg/mL after MPEP+CD treatment (4.5 g MPEP+25 mg CD; ~225 mg of LD), and 22,220 pg/mL after LD+CD administration (250/62.5).

#### 2.2.2. Experiment 1: Effects of MPEP, MPEP+CD, and LD+CD

After finding preliminary optimal doses of MPEP, we examined the effects of 4.5 g MPEP alone (~225 mg LD) (*N* = 5), 4.5 g MPEP (~225 mg LD) + 25 mg CD (*N* = 6) and 100–200 mg LD + 25–50 mg CD (i.e., daily doses were 9 g MPEP alone (*N* = 5), 9 g MPEP+50 mg CD (*N* = 6), and 200–400 mg levodopa + 50–100 mg CD (*N* = 6)) in HP and OHP primates. In a subset of animals, cranial recording chambers were surgically implanted to permit chronic single-cell extracellular neuronal recording from the left STN and SNR in the stable HP state (*N* = 3), with LD+CD treatment (*N* = 2) and with MPEP+CD treatment (*N* = 1) (a portion of this study has been presented elsewhere [16]).

#### 2.2.3. Expriment 2: Effects of MPWE and LD

MPEP was dissolved in sterile water and thoroughly mixed for 30–45 mins. The mixture was then centrifuged at 14,000–15,000 RPM for 15–20 mins, and the supernatant was extracted and filtered. The MPWE solution was then stored in sterile containers at 4°C. Prior to administration, the MPWE solution was briefly agitated and dispensed orally. The dosage concentration of MPWE was based on 4-5% of natural occurring LD in MPEP such that the MPWE solution had approximately 24 mg LD per mL.

HP, OHP, and bilateral parkinsonian animals (*N* = 5) were treated with placebo, LD+CD or MPWE b.i.d. for 3–11 days. mUPDRS scores were obtained on placebo, oral LD (1 to 3.5 tablets LD/CD–100/25), and escalating doses of 4 mL–36 mL MPWE orally (~96 mg–864 mg LD, resp.) until optimal doses were found using a blinded, randomized design with 2 weeks of washout between treatments. When compliance was an issue with oral LD+CD, animals received systemic injections of LD at the equivalent optimal doses of oral LD (LD methyl ester with benserazide (BZ)). Drug-induced dyskinesias were assessed using the abnormal involuntary movements (AIMS) rating scale in animals that displayed clear LD-induced dyskinesias similar in advanced PD [15, 18, 19]. Data was analyzed using ANOVA with Tukey posttest or Chi-square test (mean ± SEM).

## 3. Results

### 3.1. MPEP and LD Effects on Parkinsonism and Basal Ganglia Electrophysiology

**Figure 1:**
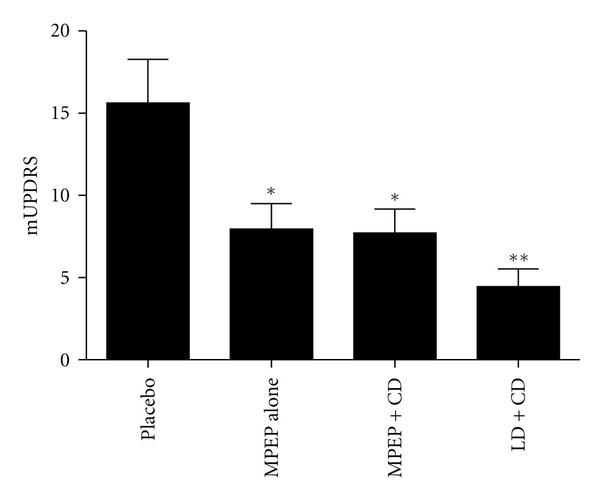
Comparison of mUPDRS scores in parkinsonian primates with placebo, MPEP alone, MPEP+CD, and LD+CD demonstrates significant amelioration of parkinsonism after treatments. *P < 0.05, **P < 0.01.

mUPDRS scores on placebo were 15.6 ± 2.6  and decreased to 8.0 ± 1.6 with MPEP alone, 7.7 ± 1.5 with MPEP+CD, and 4.5 ± 1.1 with LD+CD, optimal doses (Figure 1). These doses caused no observable adverse effects.

**Figure 2:**
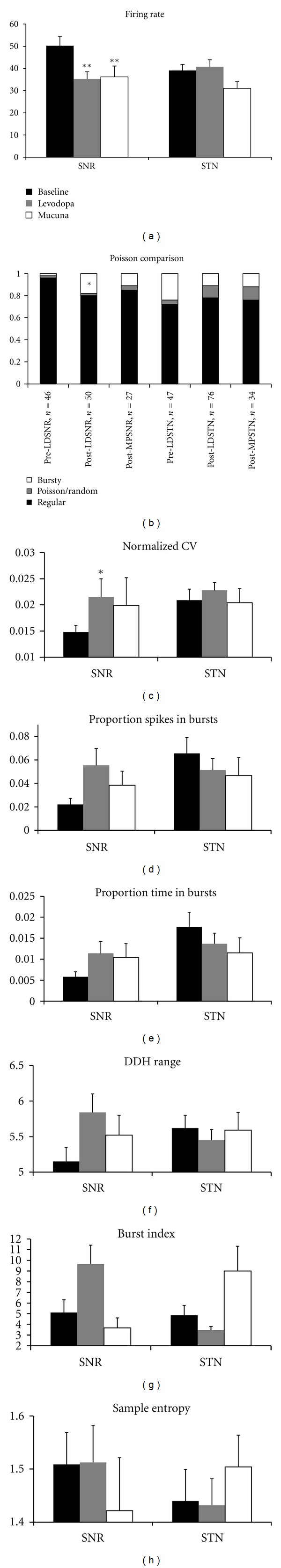
(a) Firing rates of SNR and STN in the HP monkey in stable HP state (baseline) and on LD+CD (Levodopa) and MPEP+CD (Mucuna) (Kruskal-Wallis *P* < 0.01, **P < 0.01 rank-sum using Tukey's HSD correction, compared to baseline HP state). (b) Poisson comparison of SNR and STN neurons Pre-LD (stable baseline HP state) (Fisher's 2 × 2 two-sided exact test grouping “Poisson” category together with “regular,” *P = 0.0164), Post-LD (LD treatments), and Post-MP (MPEP+CD). (c) Coefficient of variation at HP state and with treatments (Kruskal-Wallis *P* < 0.05, *P < 0.05 rank-sum using Tukey's HSD correction, compared to baseline HP) (d–g) Measures of firing patterns in the SNR and STN in HP state and on treatments. (h) Sample entropy of SNR and STN.

A portion of this electrophysiological data has been presented in a previously published report [16]. SNR firing rate showed significant reduction in SNR on both LD+CD and MPEP+CD. STN firing rate showed no significant difference, but a trend toward reduction on MPEP+CD (Figure 2(a)). SNR firing pattern became more bursty on LD+CD, measured by Poisson DDH comparison. SNR patterns changes on MPEP+CD did not reach statistical significance, but showed a trend toward increased burstiness, but not as pronounced as LD+CD. STN patterns did not show a statistical change, although both LD+CD and MPEP+CD showed a trend toward reduction of the number of bursty neurons (Figure 2(b)). Median SNR normalized coefficient of variation was higher on LD+CD than baseline HP state. On MPEP, the SNR showed a trend toward increased normalized CV, but it was not significant. There were no significant changes in the STN (Figure 2(c)). The proportion of spikes in bursts and proportion of time in bursts (measured from the Poisson-surprise method) did not show any statistically significant changes. However, there was a trend for both LD+CD and MPEP+CD to make the SNR more bursty, with MPEP+CD showing a smaller effect than LD+CD. There was a trend for LD+CD to make the STN less bursty, and MPEP+CD showed the same trend to an even larger degree (Figures 2(d) and 2(e)). DDH range counts were not statistically different in the different conditions. However, there was a trend for LD+CD to make the SNR more bursty. MPEP+CD showed a trend toward making the SNR more bursty but it was not significant (Figure 2(f)). The burst index was not statistically significant between groups. However, there was a trend for LD+CD to make the SNR more bursty, which was not replicated on MPEP+CD. In fact, there was a trend for MPEP+CD to reduce the burstiness of SNR. In the STN, the trends were reversed, but again neither were significant (Figure 2(g)). Sample entropy did not show any significant differences, although the MPEP+CD treatment slightly reduced the SNR sample entropy and slightly increased the STN sample entropy (Figure 2(h)).

### 3.2. MPWE Ameliorates Parkinsonism without Causing Drug-Induced Dyskinesias

**Figure 3:**
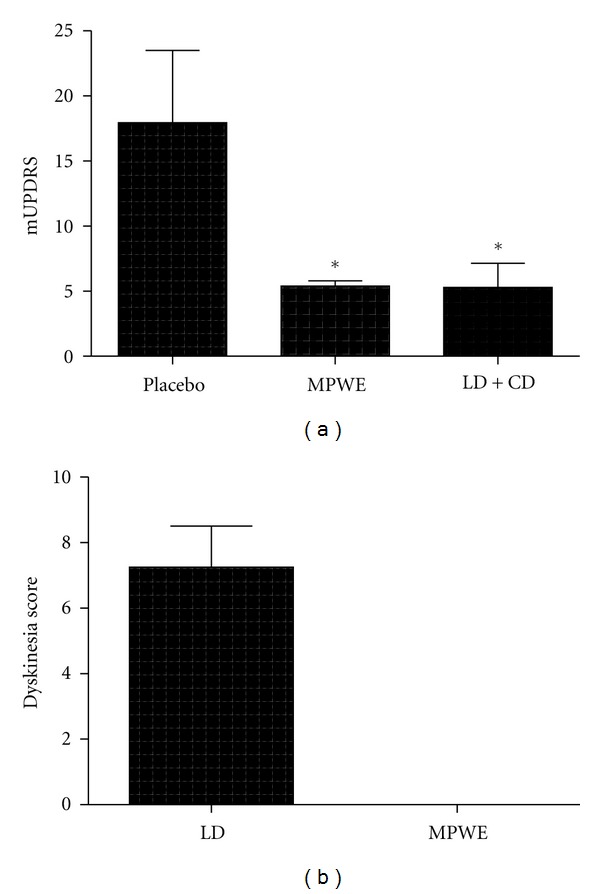
(a) A water extract of MPEP (MPWE) significantly reduces parkinsonism in the parkinsonian primate at optimal doses similar to LD+CD (*P < 0.05) (b) and does not cause dyskinesias (*P* = 0.045, Chi-square test).

mUPDRS scores on placebo were 18.0 ± 5.6, which significantly decreased with optimal doses of MPWE treatments (5.4 ± 0.4) and LD+CD treatments (5.3 ± 1.9) (Figure 3(a)). Average optimal dose of LD was 250 mg and optimal dose of MPWE was 20 mL (~480 mg LD) b.i.d. in this experiment that included HP, OHP, and bilateral parkinsonian animals. MPWE caused no apparent GI problems or drug-induced hallucinations. However, LD+CD treatments produced significant drug-induced dyskinesias (AIMS score = 7.3 ± 1.3) in two bilaterally parkinsonian animals, whereas no apparent drug-induced dyskinesias were observed with MPWE treatments (AIMS score = 0) (Figure 3(b)).

## 4. Discussion

In the present study, we demonstrate that *Mucuna pruriens *in powder and water extract form can significantly ameliorate behavioral deficits in primate models of PD. We also demonstrate that the mechanistic actions of *Mucuna pruriens *cannot be attributed to LD alone and that *Mucuna pruriens *has a unique mechanism of action on the basal ganglia electrophysiology that is different from that of LD when tested at equivalent doses. This is a confirmation of earlier suggestions that the anti-PD effects of *Mucuna pruriens *were not simply due to natural LD. Indigenous medicines based on natural products like *Mucuna pruriens *are often unique in that they contain several constituents in combination. *Mucuna pruriens *has over 50 known constituents that have been identified to date (Table 3) and perhaps others yet to be discovered [24–26]. Identifying the single component or combination of components in *Mucuna pruriens *responsible for its anti-parkinsonian/anti-dyskinetic effects is daunting. Although identification of each individual component and its exact quantity required to reproduce these effects is theoretically possible, such a task is time consuming and expensive. *Mucuna pruriens *is widely farmed in many countries as an intercrop and is exceedingly inexpensive to produce as a standardized natural product with uniform efficacy. Thus, this renewable, natural product may represent a new treatment that is different from contemporary drug discovery methods where identification of active ingredients, synthetic manufacture, safety, and efficacy testing followed by mass marketing of the synthesized compounds is replaced by a strategy that focuses on identification of safety and efficacy of a standardized natural product and its mechanism of action when used as a whole. While such an approach may sound counterintuitive, archaic, and confrontational to the current wisdom of scientific advancement, it is pragmatic and has the potential to advance the therapy of PD with the possibility of worldwide availability of inexpensive *Mucuna pruriens *formulations.

### 4.1. Effects of Mucuna pruriens Endocarp Powder

In experiment 1, we found that MPEP had to be dosed at higher quantities to get maximal effect when compared to LD (100–200 mg synthetic LD versus 225 mg natural LD in MPEP). The large volume of MPEP powder (6 g to 18 g/day) in preliminary studies and in experiment 1 was very difficult to successfully administer in monkeys due to gastrointestinal side effects, similar to those of earlier reports [1, 8–10]. These gastrointestinal effects could be in part due to the large protein content in this leguminous cotyledon powder, a well-known cause of abdominal bloating, flatulence, and gastrointestinal irritability. Serum dopamine measurements demonstrate that bioavailability and peak plasma pharmacokinetics of natural LD contained in MPEP and synthetic LD are quite similar. This finding further strengthens the notion that MPEP contains additional anti-PD and anti-dyskinetic agents beyond the 4-5% natural LD content.

Previous reports have demonstrated that LD and other dopamine replacement therapies can significantly alter firing properties of basal ganglia nuclei. This has been discussed in our recently published paper in detail [16, 20, 27–34]. We demonstrate that SNr firing rate is significantly decreased after treatment with both LD and MPEP. However, LD treatment did not decrease STN firing rate. Interestingly, MPEP did cause a trend in decreasing STN firing rate. We also found differential firing patterns between the two treatments. LD caused a significant increase in SNr bursting activity but this increase was not seen with MPEP. We also found slight differences between LD and MPEP in the other measures of burstiness. Various pharmacological agents are known to alter basal ganglia firing patterns, which include serotonin, N-methyl-D-aspartate modulators, and dopamine agonists [29, 35–37]; the presence of similar compounds in MPEP may account for the differential bursting firing patterns of MPEP.

### 4.2. Effects of Mucuna Pruriens Endocarp Powder Water Extract

The ameliorative effects of oral MPWE treatments are similar to the anti-PD effects of LD+CD treatment, a gold standard for pharmacological therapeutic efficacy in PD, sans its deleterious side effects in the parkinsonian primate. As shown in experiment 2, MPWE provides a simple and inexpensive solution to these problems with gastrointestinal intolerance of MPEP and demonstrates that the anti-PD and anti-dyskinetic compounds contained in*Mucuna pruriens* are water soluble and effective without the need for concomitant DDCI. This suggests that even monkeys “primed” to develop drug-induced dyskinesias from repeated exposure to LD+CD treatments can be successfully treated with MPWE without causing dyskinesias. Furthermore, the anti-PD and anti-dyskinetic effects of MPWE were not diminished by chronic exposure, drug washout, and reexposure. We have recently shown that LD does not cause dyskinesias in HP rhesus monkeys [15]. In this context, we demonstrate the anti-PD properties of MPWE in HP and OHP primates, models that represent restricted nigrostriatal dopaminergic loss, and the anti-PD and anti-dyskinetic effects in the bilateral parkinsonian monkey, a model that represents more severe PD and readily exhibits drug-induced dyskinesias. These findings in the MPTP-treated monkey models are similar to what we have reported in the HP rat [12]. Some investigators have argued that drug-induced abnormal involuntary movements displayed by the parkinsonian rat are not equivalent of drug-induced dyskinesias seen in PD and believe that the phenomenology of drug-induced dyskinesias in the primate model more closely resembles clinical drug-induced dyskinesias [38]. We address this in the current study, confirming the preclinical relevance of the anti-PD and anti-dyskinetic effects of MPWE without a DDCI in parkinsonian primates. Taken together, these studies in the rodent and primate models of PD provide compelling preclinical evidence of the efficacy and safety for MPWE. Biochemical measurements previously mentioned provide proof that LD+CD treatments were appropriately dosed in animals that developed drug-induced dyskinesias in the MPWE experiments. We hypothesize that the improved safety profile of MPWE may be due to additional beneficial compounds as speculated in previous studies [8, 25, 26].

We escalated the dose of MPWE more than what was needed to match the optimal anti-PD effects obtained from LD+CD treatments (MPWE doses up to >1600 mg LD equivalent dose per day). Nonetheless, these animals tolerated these large doses without adverse effects. MPWE contains 4-5% LD that is identical to the 4-5% LD content reported for MPEP powder. Since MPWE was administered without a DDCI, we hypothesize that the effects cannot be entirely due to LD content in MPWE because LD would be metabolized via peripheral DDC, suggesting that MPWE may have DDCI-like activities or other mechanisms to protect LD degradation via the action of peripheral DDCI. However, our previous rodent studies suggest that MPWE action cannot be accounted for just on the basis of its purported DDCI-like activity [12]. Thus, our rodent studies [12] and the current experiment collectively provide behavioral evidence that the anti-PD effects of MPWE cannot be explained by the presence of 4-5% natural LD alone or the combination of natural LD and a yet-to-be identified DDCI constituent. Other water-soluble compounds that remain unidentified contained in MPWE have to be implicated for the anti-PD and anti-dyskinetic effects observed in these studies. 

### 4.3. Oral Administration of Antiparkinsonian Treatments to Parkinsonian Monkeys

This is the first report of any phytomedicine that has been tested in primates using operant conditioned methods for oral voluntary consumption to simulate clinical PD pharmacotherapy, using placebo controls and a blinded prospective study design. This study design could represent an ideal method to perform future preclinical studies of phytomedicines in PD. We found compliance with oral consumption easier with MPWE compared to MPEP, MPEP+CD, or LD+CD. Previous studies with various MPEP formulations [8–10, 39, 40] have a number of disadvantages that include variable behavioral assessments, use of concomitant medications, inadequate washout, lack of LD dose controls, and excess variability in study populations (see detailed discussion in our recent report [12]). In the present study, we overcame these disadvantages by (1) using well-established primate models of PD that exhibit motor fluctuations and drug-induced dyskinesias that closely resemble its phenomenology to patients with PD, (2) ensuring drug compliance to replicate the clinical experience of PD patients, (3) utilizing the same behavioral rater for all mUPDRS assessments to eliminate interrater variability, and (4) ensuring that animals received no concomitant medications.

## 5. Conclusion

We demonstrate that *Mucuna pruriens *and MPWE have unique mechanistic properties that are differential from LD and that the unique combination of constituents within *Mucuna pruriens *contributes to both its anti-PD and anti-dyskinetic effects. This will be advantageous to PD patients who currently take LD-containing formulations and have to experience its long-term side effects that often require invasive surgical intervention. This study also shows that MPWE contains a yet-to-be investigated portfolio of anti-PD and anti-dyskinetic agents that could open up new therapeutic avenues for PD, yet constitute a daunting and expensive conventional drug discovery approach. While additional scientific studies to identify these individual anti-PD and anti-dyskinetic components contained in MPWE may be warranted, parallel studies to evaluate the clinical use of MPWE as a safe and effective alternative to LD therapy in PD is also immediately indicated with our demonstration of its unique beneficial mechanisms of action.

## Figures and Tables

**Table 1:** Conditions for operant conditioning in parkinsonian primates.

Ideal conditions for training	Ideal conditions for enrichment
Single-housed animals	Group-housed animals
Supplemental toys without food (mirrors, chew toys)	Supplemental toys containing food (foraging devices)
No visual contact with conspecifics	Visual contact with conspecifics
No sound	Sound (movies, radio, wildlife sounds)

**Table 2:** Dosing regimen for non-human primates. A block design was devised for each testing session. Each behavioral testing cohort was varied to maintain blind and to prevent the behavioral rater from guessing the treatments. Each such block design was repeated twice for each experiment and videos are independently rated. Representative animals were videotaped continuously in the room 24 × 5 × 365 days. The postdrug treatment videos were culled from these videos by the person who administered the drug who was unblinded. These culled video segments were used for the scoring along with the rater executed direct observational scoring of mUPDRS and AIMS. These culled segments began as soon as the person administering the drug confirmed successful consumption of the drug and lasted 8 hours from that time. The notion of the average time of 75 minutes refers to the average time at which behavioral ratings using mUPDRS and AIMS were scored for the study. The validity of this time frame for the detection of optimal effects of LD and LD/DDCI oral treatments has been published previously. To make meaningful valid comparisons MPEP and MPWE treatments were also performed at the same time schedule. The remainder of the video was rated, but it does not have the observer interaction and it only shows routine animal activity in its home cage. As expected, LD and LD/DDCI treatments had behavioral benefits that lasted 180 minutes and then ameliorated. Effects of MPEP and MPWE lasted longer and appeared to dissipate only after 6 hours.

	Monkey 1	Monkey 2	Monkey 3	Monkey 4	Monkey 5
Block 1	LD	Placebo	Placebo	MPEP	MPEP
Block 2	LD	Placebo	Placebo	MPEP	MPEP
Block 3	Placebo	LD	LD	Placebo	Placebo
Block 4	Placebo	LD	LD	Placebo	Placebo
Block 5	MPEP	Placebo	Placebo	LD	LD
Block 6	MPEP	Placebo	Placebo	LD	LD
Block 7	Placebo	MPEP	MPEP	Placebo	Placebo
Block 8	Placebo	MPEP	MPEP	Placebo	Placebo

**Table 3:** Known components of *Mucuna pruriens*.

Arachidic acid	Lysine
Arginine	Methionine
Ash	6-Methoxyharman
Aspartic acid	1-Methyl-3-carboxyl-6,7-dihydroxy-1,2,3,4-tetrahydroisoquinoline
Behenic acid	Mucunadine
Beta carboline	Mucunain
Beta sitosterol	Mucunine
Bufotenine	Myristic acid
Calcium	Niacin
Carbohydrates	Nicotine
Choline	Nicotinamide adenine dinucleotide
Cystine	Oleic acid
Coenzyme Q-10	5-Oxyindole-3-alkylamine
N,N-Dimethyltryptamine	Palmitic acid
N,N-Dimethyltryptamine-N-oxide	Palmitoleic acid
L-Dopa	Phenylalanine
Cis-12,13-epoxyoctadec-trans-9-cis-acid	Phosphorus
Cis-12,13-epoxyoctadec-trans-9-enoic-acid 5-Methoxy-N,N-dimethyltrytamine-N-Oxide	Proline
Fat	Protein
Fiber	Prurienidine
Gallic acid	Prurienine
Glutamic acid	Riboflavin
Glutathione	Saponins
Glycine	SD
Histidine	Serine
5-Hydroxytryptamine	Serotonin
Indole-3-alkylamine	Stearic acid
Iron	Thiamin
Isoleucine	Threonine
Lecithin	Tryptamine
Leucine	Tyrosine
Linoleic acid	Valine
Linolenic acid	Vernolic acid
